# Supplementary material for: Identification of photoperiod-induced specific miRNAs in the adrenal glands of Sunite sheep (Ovis aries)
Source: Front Vet Sci. 2022 Jul 22;9:888207. doi: 10.3389/fvets.2022.888207 (PMC9354845; doi:10.3389/fvets.2022.888207)
Supplement: Supplementary file 1 [file Data_Sheet_1.ZIP › Supplementary file/miRDeep2 file.zip!/miRDeep2 file/Novel_Structure/SPLP42A1/Novel_374.pdf]

Provisional ID	:	NC_019459.2_3596
Score total	:	2.4
Score for star read(s)	:	-1.3
Score for read counts	:	0
Score for mfe	:	2.1
Score for randfold	:	1.6
Score for cons. seed	:	
Total read count	:	139
Mature read count	:	130
Loop read count	:	0
Star read count	:	9

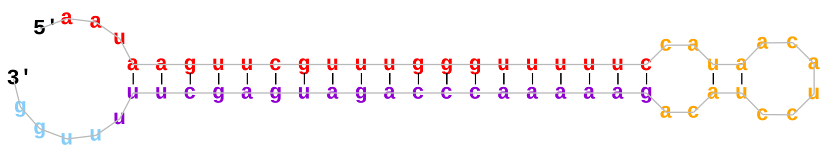

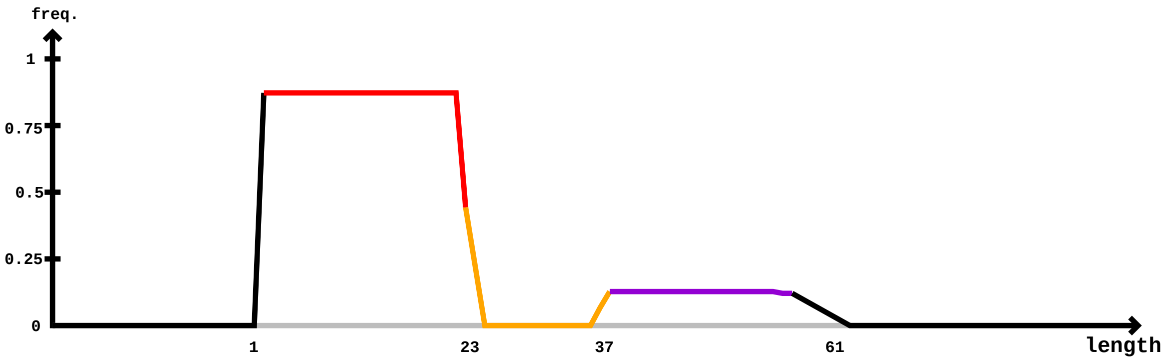

Mature Star

```
5'- aaauacauguuggaguggccaauaaguucguuuggguuuuuccauaacauccuacagaaaaacccagaugagcuuuuuggccaacccaauacuuuauuuccuuuucgggguc   -3'   obs
    aaauacauguuggaguggccaauaaguucguuuggguuuuuccauaacauccuacagaaaaacccagaugagcuuuuuggccaacccaauacuuuauuuccuuuucgggguc         exp
    .......((((((..(((((((.((((((((((((((((((((..((......))..)))))))))))))))))))).))))))))..)))))).........(((....)))..   reads   mm   sample
    ....................aauaaguucguuuggguuuuu......................................................................   2   0   seq
    ....................aaAaaguucguuuggguuuuu......................................................................   62   1   seq
    ....................aaAaaguucguuuggguuuuuc.....................................................................   66   1   seq
    .......................................................agaaaaacccagaugaAcuuu...................................   3   1   seq
    .......................................................agaaaaacccagaugaAcuuuu..................................   7   1   seq
    ........................................................gaaaaacccagaugaAcu.....................................   1   1   seq
    ........................................................gaaaaacccagaugaAcuuu...................................   8   1   seq
```
